# Pneumocystis Pneumonia in Previously Undiagnosed Advanced HIV/AIDS and Importance of HIV Screening

**DOI:** 10.7759/cureus.69902

**Published:** 2024-09-22

**Authors:** Mounica Boggala, Muhammad Junaid, Elizabeth Zhang

**Affiliations:** 1 Medical School, Lincoln Memorial University - DeBusk College of Osteopathic Medicine, Knoxville, USA; 2 Internal Medicine, Methodist Le Bonheur Healthcare, Memphis, USA

**Keywords:** acquired immune deficiency syndrome (aids), hiv/aids related opportunistic infections, hiv diagnosis, pneumocystis jirovecii pneumonia, universal hiv screening

## Abstract

The diagnosis of human immunodeficiency virus (HIV) in its late stages, also known as acquired immunodeficiency syndrome (AIDS), leads to increased morbidity and mortality. This is mostly due to the time given for opportunistic infections to arise, which present with their own complications. In this case report, we present an otherwise healthy 38-year-old male, who presented with general systemic symptoms and was later found to have HIV and AIDS, ultimately resulting in his death during this hospital admission. This case report will discuss HIV-associated opportunistic infections, focusing more on* Pneumocystis jirovecii *pneumonia (PCP), and the following complications that arose during the course of this patient’s hospitalization. The overarching goal of this case report is to highlight the importance of routine HIV screening in the United States in efforts to decrease mortality from AIDS-related opportunistic infections. In addition to mandating in-hospital screening, educating the public on lifestyle behaviors that can put one at risk for developing HIV is crucial to decreasing the prevalence of cases that go undiagnosed, as well as the disease itself.

## Introduction

Delayed HIV diagnosis leads to higher mortality and morbidity rates [1]. According to data collection from 2018, 14% of HIV cases in the United States remain undiagnosed [2]. This means that the individuals carrying their unknown disease can progress to AIDS, the third and latest stage of HIV. There are three distinct stages of the progression of HIV. During the first stage, also known as the acute phase, the virus replicates rapidly and the individual may experience generalized systemic symptoms, including fever, fatigue, and myalgias [3]. The next stage is symptomatically silent in the majority of cases, as the virus continues to replicate, but more slowly than in the first stage [3]. The last and final stage of HIV is what we know as AIDS. AIDS is defined by a cluster of differentiation four (CD4) count of less than 200 cells/mm^3^. CD4+ T helper cells play a role in our body’s innate and adaptive immune system, providing a response against various pathogens which can infect us [4]. This stage begins to take a large toll on the individual’s immune system, as CD4 cells start to drop, putting the individual at high risk for developing several opportunistic infections. It is crucial to initiate antiretroviral therapy as soon as the diagnosis of HIV is made in order to prevent the later stages of the disease from developing [5].

The most commonly known opportunistic infections with antimicrobial prophylaxis that may present with a diagnosis of AIDS are *Pneumocystis jirovecii*, *Toxoplasma gondii*, *Coccidioides*, and *Mycoplasma tuberculosis* [6]. Other infections include *Cryptosporidium*, *Mycobacterium avium* complex, *Histoplasma*, and *Candida*. Each of these is most prevalent below a specific CD4 count. It is indicated to screen for these opportunistic infections once an individual with HIV has a CD4 count that is less than 200. Prophylactically treating these infections is important to avoid poorer outcomes. Prophylaxis can be discontinued when the CD4 rises back up to greater than 200. *Pneumocystis jirovecii* is the most common opportunistic infection arising from AIDS, and also one of the most fatal [7]. PCP accounts for roughly 10,000 hospitalizations in the United States alone per year, and more than 400,000 in the world [8]. Medical therapy for this infection has primarily been for the prevention of the disease using trimethoprim-sulfamethoxazole (TMP/SMX). This case report discusses the presentation of PCP in a patient who presented with a CD4 count of 20 with previously undiagnosed HIV.

## Case presentation

A 38-year-old African-American male with a past medical history of hypertension presented to the emergency department with ongoing symptoms of nausea, vomiting, dizziness, and shortness of breath for a few weeks. He reported emesis to both solid and liquid foods and was febrile for three days prior to admission. On arrival, his vitals were: Oral temperature 37.4℃, blood pressure 183/100, pulse 125 bpm, respirations 20, oxygen 100% on room air. Labs were notable for the following (Table 1):

**Table 1:** Notable lab values on arrival to the emergency department. INR: International normalized ratio

Lab Values	Patient	Reference Range [9,10]
Sodium (mEq/L)	131	136–145
Potassium (mEq/L)	2.7	3.5–5.0
Calcium (mg/dL)	7.8	8.6–10.2
Glucose (mg/dL)	108	70–99
Creatinine, serum (mg/dL)	9.16	male: 0.70-1.30
Phosphorus (mg/dL)	6.4	3.0–4.5
Uric acid (mg/dL)	16.4	3.0–7.0
Lactic acid (mmol/L)	2.2	0.7–2.1
Hemoglobin (g/dL)	5.8	male: 14–18
Hematocrit (%)	17.4	male: 42–50
INR	1.2	0.9-1.2

**Figure 1:**
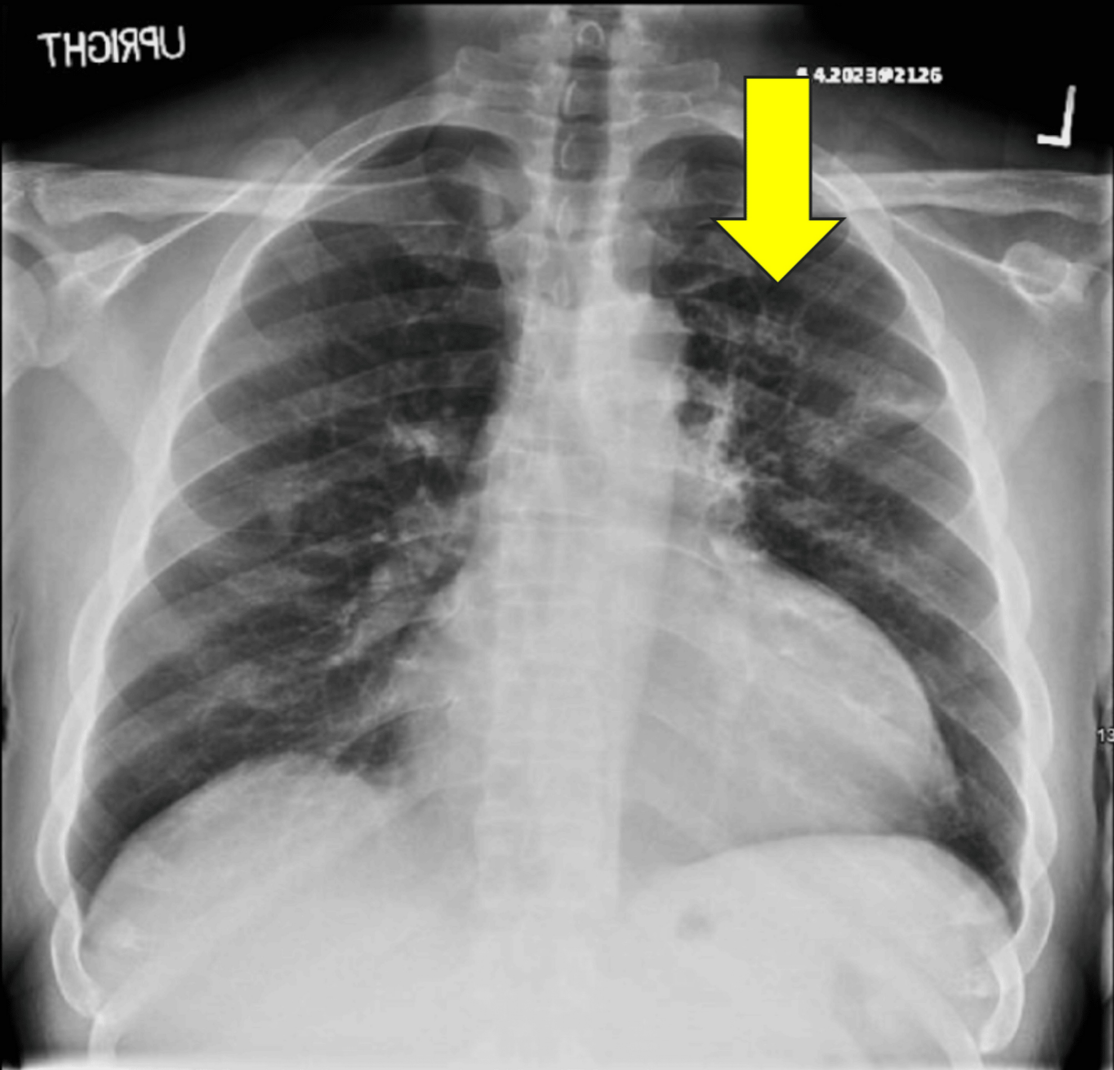
Anteroposterior view of chest X-ray demonstrating patchy patchy airspace disease in the left upper lobe.

**Figure 2:**
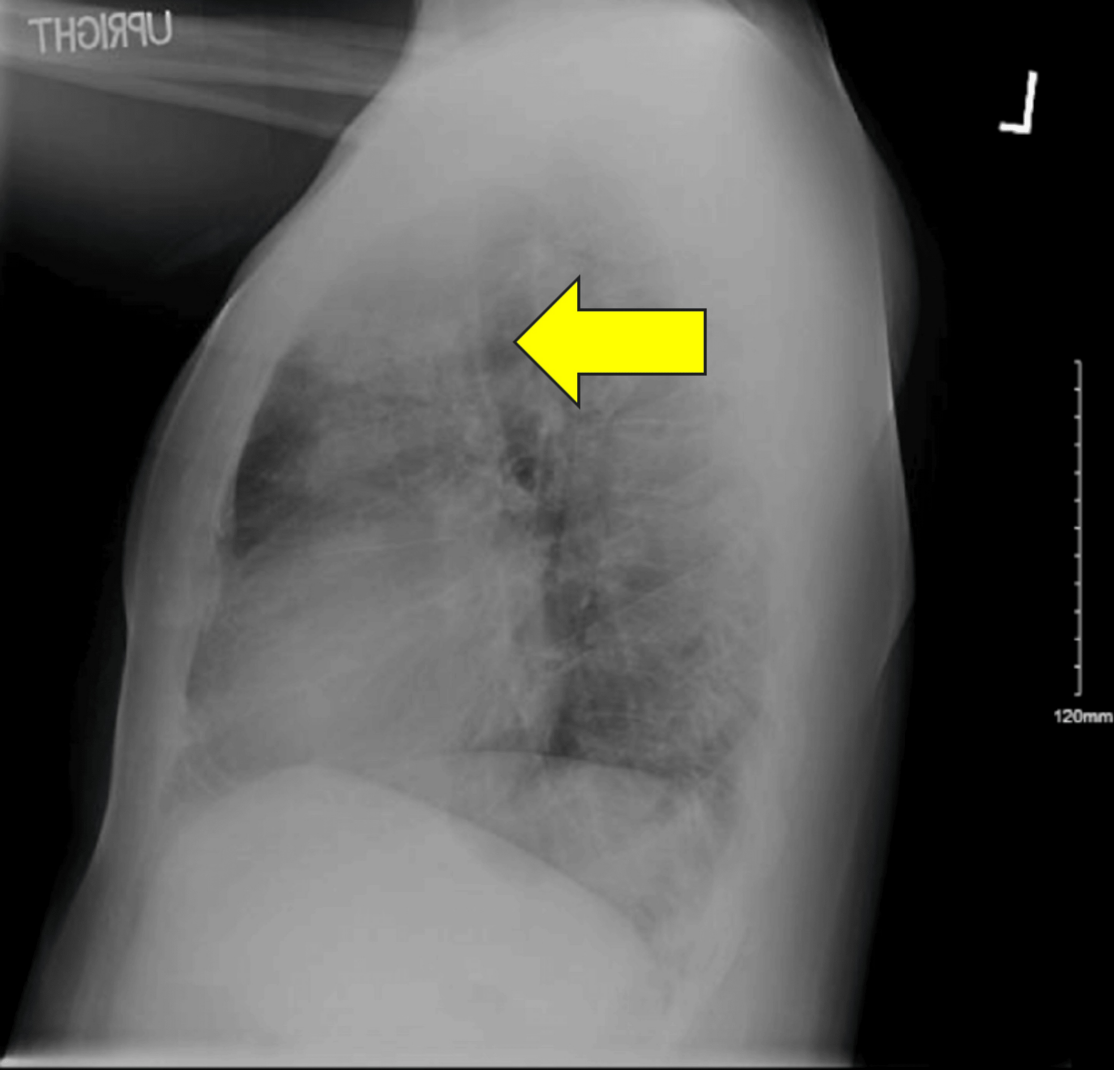
Lateral view of chest X-ray demonstrating patchy airspace disease in the left upper lobe.

The physical exam was largely unremarkable, although notable for an S4 upon cardiac auscultation. A chest X-ray demonstrated patchy airspace disease within the left upper lobe with no pleural effusion or pneumothorax identified (Figures 1, 2).

Upon routine hospital screening, the patient was found to be positive for HIV with a CD4 count of 20, indicative of AIDS, prompting an infectious disease consultation. With a low CD4 count, concern for opportunistic infections was warranted, especially given his chest X-ray findings. While awaiting his infectious disease workup, nephrology and hematology were consulted due to concern for thrombotic microangiopathy. They initiated plasmapheresis on the second day of hospitalization. A concern for possible focal segmental glomerulosclerosis led to a renal biopsy complicated by a perinephric hematoma, which was embolized by the interventional radiology team. The bronchoalveolar lavage was positive for *Pneumocystis jirovecii* and acid-fast bacilli (AFB), indicative of a *Mycoplasma* infection. The medical team made the decision to prioritize the treatment of his PCP, followed by initiation of highly active antiretroviral therapy as an outpatient. The patient was started on TMP/SMX and a course of prednisone for his PCP. For his *Mycoplasma* and possible disseminated *Histoplasmosis*, given his other infections, he was started on micafungin. He was also initiated on vancomycin after blood cultures grew *Staphylococcus capitis*. Several days into his hospitalization, he experienced new onset of bloody diarrhea. Gastroenterology (GI) was consulted, and they started him on azithromycin for possible *Escherichia coli* colitis.

On the tenth day of his hospitalization, the patient’s temperature rose to 39℃, pulse was 109 bpm, respiratory rate was 32, blood pressure was 58/34, and oxygen saturation was 92%. His mental status declined and he became unresponsive during the evaluation. He was transferred to the intensive care unit and started on Levophed. Code blue was called, however, it was unsuccessful. The patient passed away later that afternoon.

## Discussion

In patients who have an unknown diagnosis of HIV or have known HIV but are not on antiretroviral therapy, the CD4 count can be severely decreased. This puts them at very high risk of HIV-related opportunistic infections. Not only is that individual at risk of disease progression, but also it is estimated that 40% of new HIV diagnoses are transmitted by those who are not aware of their diagnosis [11], posing as a risk to others who may also be involved in high-risk behaviors. As discussed previously, Pneumocystis jirovecii pneumonia can occur in those with a CD4 count of <200 cells/mm^3^. Patients with PCP may present with progressive dyspnea and fever [12]. This fungal infection can cause pneumonia that may be life-threatening [13]. A chest X-ray would demonstrate diffuse interstitial infiltrates in a ground glass pattern [12]. To confirm the diagnosis of PCP, as we discussed in the Case Presentation, we would obtain a bronchoalveolar lavage to directly assess the fluid. Other methods include polymerase chain reaction (PCR), antibody-antigen assays, and loop-mediated isothermal amplification (LAMP), which are also sensitive methods to detect PCP [13]. Prophylactic treatment for PCP entails the initiation of trimethoprim/sulfamethoxazole (TMP/SMX) [8].

Overall PCP has high mortality rates with rates actually being higher in individuals with no underlying diseases compared to those who are immunocompromised. A study done in 2021 also showed that delaying treatment for PCP resulted in a higher 90-day mortality rate [14]. The same study showed that treatment for PCP in HIV patients compared to non-HIV patients was more important for survival outcomes [14]. Therefore, delayed PCP treatment is a risk factor for mortality. This leads us to discussing the importance of screening for HIV which would then lead us to clues for opportunistic infections based on patients’ symptoms after obtaining a CD4 count. As we observed in our case, our patient had developed PCP in the setting of unknown HIV status prior to his admission. Although we proceeded with HIV screening and treatment of his PCP upon hospitalization, if he was diagnosed even earlier it would have benefited him more. The lack of early initiation of treatment given his unknown HIV status, along with other complications he faced surrounding his disease, resulted in his death.

In this context, the “Centers for Disease Control and Prevention” (CDC) recommends that all individuals from ages 13 to 64 be screened at least once as general health care, while high-risk populations should receive a minimum of one screening test each year [8,11]. The National HIV Strategic Plan for the US recommends doing routine HIV screening in emergency departments [15]. The main groups that have been recognized as “high risk” are intravenous drug users (IVDUs), sex workers, men who have sex with men (MSM), sex partners of HIV-infected persons, and partners of at-risk subjects [8]. However, the majority of the US states do not execute HIV testing that is consistent with these recommendations. Furthermore, there are limited literature reports about universal HIV screening, particularly regarding emergency departments. Although the prevalence of unknown HIV status is decreasing, a 2011 study reported that 75% of patients presenting to an emergency department aged 13 years and older still had unknown HIV status [16].

There is growing evidence regarding the health benefits and cost-effectiveness of HIV screening, with the major determinant of cost-effectiveness being the baseline HIV prevalence in a particular region [8]. Repeat testing should be performed in high-risk groups, but even one-time screening for the general population is worthwhile. Both standard and rapid testing have been shown to be cost-effective [8]. Screening methods include antibody tests, antigen/antibody tests, and nucleic acid tests [11]. If the initial test is positive, a follow-up test is done to either conclude a false-positive result or confirm an HIV diagnosis. From this case report, we demonstrate how a patient would have benefited from an earlier diagnosis with routine HIV screening. It also demonstrated how HIV screening inpatient during this admission led us to our diagnosis of AIDS complicated by PCP and *Mycoplasma* infections.

## Conclusions

HIV/AIDS remains to be a widely stigmatized diagnosis worldwide, oftentimes causing a lack of knowledge in preventive strategies and delay in treatment. Without initiating treatment in a timely manner, the prognosis continues to be poor. This case demonstrated the rapid decline of a previously overall healthy 38-year-old male who did not develop any symptoms of his disease until it was too late to be medically reversible. Therefore, this case should serve as an eye-opener for the need to screen patients for HIV along with all the other normalized routine tests we tend to do in the hospital as well as in outpatient settings. It is crucial to test patients for HIV at least once a year as well as at every hospital admission to prevent the late-stage diagnosis of HIV/AIDS and the outcomes of some of the most fatal opportunistic infections.
